# The Fsr transporter of Sinorhizobium meliloti contributes to antimicrobial resistance and symbiosis with alfalfa

**DOI:** 10.1099/mic.0.001566

**Published:** 2025-05-21

**Authors:** Victor M. Chávez-Jacobo, Alma R. Reyes-González, Lourdes Girard, Michael F. Dunn

**Affiliations:** 1 Departamento de Microbiología Molecular, Instituto de Biotecnología, Universidad Nacional Autónoma de México, Avenida Universidad 2001, Cuernavaca, Morelos, Mexico; 2 Programa de Genómica Funcional de Procariotes, Centro de Ciencias Genómicas, Universidad Nacional Autónoma de México, Avenida Universidad s/n, Cuernavaca, Morelos, Mexico; 3 Programa de Microbiología Genómica, Centro de Ciencias Genómicas, Universidad Nacional Autónoma de México, Avenida Universidad s/n, Cuernavaca, Morelos, Mexico

**Keywords:** antibiotic efflux pumps, antibiotic resistance, biofilm formation, fosmidomycin, *Sinorhizobium meliloti*–alfalfa symbiosis, swimming motility

## Abstract

Major facilitator superfamily (MFS) transporters in bacteria participate in both the uptake and export of ions, metabolites or toxic compounds. In rhizobia, specific MFS transporters increase resistance to plant-produced compounds and may also affect other phenotypic traits, including symbiosis with legume host plants. Here, we describe the importance of the *Sinorhizobium meliloti* 1021 Fsr efflux pump in resistance to selected antimicrobial compounds and in modulating biofilm formation, motility and symbiotic efficiency with alfalfa. The *fsr* gene (*smc00990*) is annotated as encoding an MFS family fosmidomycin efflux pump. Unexpectedly, both the 1021 wild type and an *fsr* null mutant were highly resistant to fosmidomycin. Our assays indicate that this is due to an inability to transport the antibiotic. Unlike the wild type, the *fsr* mutant was highly sensitive to the fosmidomycin structural analogue fosfomycin, and moderately more sensitive to hydrogen peroxide (H_2_O_2_) and deoxycholate (DOC). Root and seed exudates from alfalfa did not inhibit the growth of the wild type or *fsr* mutant. *fsr* transcription significantly increased proportionally to the concentration of fosfomycin added to cultures but was unaffected by the addition of other antibiotics, H_2_O_2_, DOC or SDS. Alfalfa seed exudate moderately increased *fsr* transcriptional expression. Fluorometric assays using ethidium bromide as a substrate and carbonyl cyanide m-chlorophenyl hydrazone as an energy decoupler showed that Fsr was a proton-dependent efflux pump. Biofilm formation and swimming motility were decreased and increased, respectively, in the *fsr* mutant, and its symbiotic efficiency with alfalfa was decreased in terms of nodule numbers per plant and plant dry weights.

## Data Availability

No additional datasets were produced in this work.

## Introduction

Multidrug resistance (MDR) efflux pumps in beneficial rhizobacteria and bacterial phytopathogens are important for resistance to toxic compounds made by host plants or other soil microbiota. MDR systems in plant-associated bacteria also affect intra- and interspecies signalling, stress responses and biofilm formation (for reviews, see references [1,4]). Most work on MDR pumps has focused on their role in bacterial antibiotic resistance. However, the genes encoding MDR systems evolved long before the use of antibiotics in medicine and agriculture, and there is no apparent correlation between a strain’s spectrum of antibiotic resistance and the number of MDR pumps it contains [2].

Most MDR efflux systems are proton-driven pumps belonging to the major facilitator superfamily (MFS). MFS pumps export a broad spectrum of substrates including antimicrobials, metabolites, ions, virulence factors, plant-produced compounds and quorum-sensing signal molecules [4,5].

MDR efflux pumps affect the efficiency of the symbiosis between nitrogen-fixing rhizobia and their legume hosts. Plants' roots secrete antimicrobial secondary metabolites that protect them against pathogen infection or participate in signalling pathways including nodule initiation in legumes [6]. Inactivation of the *Rhizobium etli* flavonoid-inducible RmrAB MFS pump increased the sensitivity of *R. etli* to flavonoids and reduced its ability to form nodules on bean roots [7]. In *Bradyrhizobium japonicum*, the resistance-nodulation-cell division (RND) superfamily pump BdeAB was not required for nodule formation on soybean but was essential for nitrogen fixation [8]. In *Sinorhizobium meliloti* 1021, the RND-type efflux pumps SmeAB, SmeCD and SmeEF were shown to be important in resistance to a number of antimicrobials, and a SmeAB mutant had lower competitiveness for nodule formation on alfalfa [9]. An * S. meliloti* 1021 deletion mutant lacking EmrR, a regulator of the EmrAB efflux system, showed decreased motility, biofilm formation and ability to attach to alfalfa roots [10].

We recently characterized the *S. meliloti* NspS-MbaA sensor/transducer system that affects motility, biofilm formation and symbiotic efficiency in response to specific polyamines. The gene arrangement surrounding the *nspS-mbaA* operon was identical in 41 out of 42 *S*. *meliloti* strains [11]. This conserved region included a gene encoding a predicted fosmidomycin resistance transmembrane protein (Fsr) located immediately upstream of *nspS*. The proximity of *fsr* to *nspS-mbaA* suggests potential physiological connections worth exploring. Given the importance of antimicrobial resistance mechanisms in rhizobial biology and plant-microbe interactions, we undertook the characterization of the *fsr* gene and its product to better understand its role in *S. meliloti*.

We show here that *fsr* in *S. meliloti* 1021 encodes a proton-dependent efflux pump that, contrary to expectations, does not confer resistance to fosmidomycin, an antibiotic that targets the enzyme 1-deoxy-d-xylulose-5-phosphate reductoisomerase (Dxr) of the non-mevalonate pathway for isoprenoid synthesis. Fsr was essential for the high level of resistance of *S. meliloti* to fosfomycin, a structural analogue of fosmidomycin that acts upon UDP-*N*-acetylglucosamine 1-carboxyvinyltransferase (MurA), which catalyses the first step in bacterial cell wall synthesis. Fsr also conferred increased resistance to a number of other antimicrobial compounds. An *fsr* null mutant was altered in swimming motility, biofilm formation and symbiotic efficiency on alfalfa.

## Methods

### Bacterial strains, plasmids and media

The bacterial strains and plasmids used in this study are listed in Table 1. Rich media (peptone-yeast extract (PY) and Luria broth (LB)) and minimal medium succinate-ammonium (MMSN) were prepared as described previously [12,13]. Antibiotics were included as needed at the following concentrations (µg ml^−1^): gentamicin (Gm), 15; kanamycin (Km), 50; tetracycline (Tc), 10; spectinomycin (Sp), 100; and streptomycin (Sm), 200.

**Table 1.** Strains and plasmids used in this study

Bacterial strains	Relevant characteristics	Source of reference
*E. coli* DH5α	Strain for cloning work and bioassay of fosmidomycin uptake	Laboratory collection
*S. meliloti* 1021	Wild type. Sm^r^ derivative of WT strain SU47, Sm^r^ Nal^r^	[42]
*S. meliloti* Rm8530	WT *expR*^+^ derivative of *S. meliloti* 1021, Sm^r^	[43]
*S. meliloti* 1021 Fsr	1021 *fsr*::loxSp, Sm^r^ Sp^r^	This study
**Plasmids**		
pBBMCS53	∆p*lacZ* pBBR1MCS-5 derivative with promoterless *gusA*, Gm^r^	[44]
pMS102loxSp17	Source of the loxP Sp interposon, Sm^r^	[45]
pBB53Fsr::gusA	Transcriptional *fsr*::*gusA* fusion in pBBMCS-53, Gm^r^	This study
pJQ200SK^+^	Suicide vector for gene replacement, Gm^r^	[46]
pK_Fsr	pJQ200SK^+^ containing the 1021 *fsr* gene, Gm^r^	This study
pJQ-Fsr::loxSp	LoxSp-interrupted *fsr* gene cloned in pJQ200SK^+^, Gm^r^ Sp^r^	This study
pRK2013	Helper plasmid, Km^r^	[47]
pTopo	pCR2.1 Topo vector for cloning PCR products, Km^r^	Invitrogen
pTopo-PRFsr	pTopo containing 113 of the *fsr* coding sequence and 112 upstream nt	This study
pTopo-Fsr	pTopo containing 1.2 kb of the *fsr* coding sequence	This study
pTopo-Fsr-2	pTopo containing 1.3 kb of the *fsr* coding sequence and 100 upstream nt	This study
pFAJ1700	Stable RK2 derived cloning vector Tc^r^	[48]
pFAJ_Fsr	pFAJ1700 containing 1.3 kb of the *fsr* coding sequence and 100 upstream nt	This study

### DNA manipulations

The cultivation of *Escherichia coli* strains, DNA isolation, restriction enzyme digests, cloning and bacterial conjugations and transformations were done by published protocols [12,13]. PCR amplifications were done using Dream Taq PCR Master Mix (Thermo Fisher, Waltham, MA, USA) with oligonucleotides synthesized by the Biotechnology Institute-UNAM, Cuernavaca, Mexico.

### Mutant construction

The *S. meliloti* 1021 *fsr* gene (GenBank accession SM_RS04390; old locus tag smc00990) was inactivated by replacing a portion of the gene with a spectinomycin resistance cassette. To do this, *fsr* was amplified with PCR primers SMc00990-F (5′- CATTCCATGGCAACGGTCACG-3′) and SMc00990-R (5′- GTTTATCCCGGGGGCAGCTTC-3′). The PCR programme consisted of an initial denaturing step at 95 °C for 3 min followed by 35 cycles of 95 °C for 30 s, 58 °C for 30 s and 72 °C for 1 min, with a final extension at 72 °C for 10 min. The 1.2 kb product was cloned into pTopo to generate plasmid pTopo-Fsr (Table 1). The insert from pTopo-Fsr was excised with *Sma*I and *Eco*RI and ligated into suicide vector pJQ200SK^+^ to give plasmid pK-Fsr. The loxSp element from pMS102loxSp17 was inserted between the *Xho*I and *Nde*I sites in *fsr*, causing a 639 nt deletion from the *fsr* sequence. The resulting plasmid, pJQ-Fsr::loxSp, was conjugated into *S. meliloti* 1021 and double recombinants selected by sucrose selection [11]. The correct construction of the resulting 1021 *fsr*::loxSp mutant was confirmed by PCR [11] and designated 1021 Fsr (Table 1).

### Construction of an *fsr* transcriptional fusion with the *β*-glucuronidase (gusA) gene

The 5′ portion of the *S. meliloti fsr* gene and its putative promoter sequence were amplified with primers pSMc00990-F1 (5′- ATGGCCCACTCACGAAAGAA-3′) and pSMc00990-R1 (5′- GTCAGCAGCGATTGCATGAT-3′). The PCR cycling programme included a 5 min denaturing step at 95 °C followed by 35 cycles of 95 °C for 30 s, 52 °C for 30 s and 72 °C for 0.5 min. A final extension at 72 °C for 10 min was made. The PCR product contained 113 nt of the *fsr* coding sequence and the entire intergenic sequence of 112 nt upstream of the *fsr* start codon. This upstream region contained the predicted AAC transcriptional start site 8 nt upstream of the *fsr* start codon [14]. The PCR product was cloned into pTopo to generate plasmid pTopo-PRFsr. The PRFsr fragment was excised from this plasmid with KpnI and XhoI and ligated into vector pBBMCS53 to obtain plasmid pBB53fsr::gusA. The correct transcriptional orientation of the fusion was confirmed by PCR using the gene-specific primers pSMc00990-F or pSMc00990-R in combination with pBBMCS53-specific primer pguslw (5′-ACAGGACGTAACATAAGGGAC T-3′). Plasmid pBB53fsr::gusA was transferred to * S. meliloti* 1021 by conjugation.

### Construction of pFAJ_Fsr plasmid for complementation

The *S. meliloti fsr* gene and its putative promoter sequence were amplified with primers pSMc00990-F1 and SMc00990-R (described above). The 1.3 kb PCR product was cloned into pTopo to generate plasmid pTopo-Fsr-2 (Table 1). The insert from pTopo-Fsr-2 was excised with *Hind*III and *Xba*I and ligated into pFAJ1700 to give plasmid pFAJ_Fsr. The correct transcriptional orientation of the insert was confirmed by restriction enzyme analysis, and the plasmid was transferred to the *S. meliloti fsr* mutant by conjugation.

### Inhibition zone assays

Inhibition zone assays were performed as described in [15]. Briefly, cultures were grown in PY overnight, and ~1×10^7^ cells of the bacterial strain to be tested were spread on PY plates containing appropriate selective antibiotics. Fifteen microlitres of neomycin (60 mg ml^−1^), kanamycin (30 mg ml^−1^), chloramphenicol (10 mg ml^−1^), deoxycholic acid (sodium salt) [10% (wt vol^−1^) (pH 6.8)], SDS [10% (wt vol^−1^)], salicylic acid [200 mg ml^−1^ (pH 6.8)], citric acid [200 mg ml^−1^ (pH 6.8)], fosmidomycin (20 mg ml^−1^) or fosfomycin (25 mg ml^−1^) or 10 µl of H_2_O_2_ (10 mM) or 10 µl of seed or root exudate concentrates were deposited on sterile 6 mm paper discs placed on the surface of the agar. The plates were incubated for 48 h, and the diameters of the growth inhibition zones were measured. Statistical analyses were performed using GraphPad Prism 8 software to determine differences between groups using the ANOVA test. Differences were considered to be statistically significant at *P*≤0.05.

### MIC assay

Antibiotic MICs for *S. meliloti* and *E. coli* strains were determined using the broth microdilution assay in PY and LB media, respectively, as described in [16].

### Drop assay to determine the effect of fosfomycin on bacterial survival

Overnight cultures of *S. meliloti* strains were used to inoculate PY medium containing 0, 50, 100 or 200 µg ml^−1^ of fosfomycin to an OD_590_ of 0.05. The cultures were grown at 30 °C with shaking, and at 0, 30, 60 and 90 min intervals, 10 µl aliquots were spotted on solid PY medium and incubated at 30 °C for 48 h.

### Fosmidomycin uptake bioassay

Fosmidomycin transport by *S. meliloti* 1021 was determined by a modification of the bioassay described by Brown and Parish [17]. As an indicator strain to detect fosmidomycin in culture supernatants and cell lysates, we used *E. coli* DH5α, which we determined to have an MIC for fosmidomycin of 4 µg ml^−1^ (results not shown). For the transport assay, strains 1021 and DH5α were grown overnight at 30 °C, 200 r.p.m. in PY and LB media, respectively. Cells obtained following centrifugation (5 min at 3,000 ***g***) were resuspended to a concentration of ~10^11^ cells ml^−1^ in Tris buffer (50 mM, pH 7.0). One millilitre of bacterial suspension was combined with 0.5 ml of fosmidomycin (1 mg ml^−1^) and 3.5 ml of PY or LB medium for strains 1021 and DH5α, respectively. Cultures were incubated at 30 °C with shaking at 100 r.p.m. for 1 h. Culture supernatants were obtained by centrifugation as described above and sterilized by passage through a 0.22 µM filter. Cell pellets were washed twice with 5 ml of 50 mM Tris pH 7 and resuspended in 1 ml of 10 mM Tris pH 7. Cells were disrupted by sonication on ice water using 5–30 s pulses with a microprobe at 12 µM power setting (MSE Soniprep, Dalton Scientific, London). Lysates were centrifuged at 16,000 ***g***, and lysate supernatants were sterilized by 0.22 µM filtration. For assay, 10 µl of cell lysate or culture supernatant was applied to paper discs on plates seeded with strain DH5α as described in the *Inhibition Zone Assays* section above. Inhibition zones were measured following 24 h incubation at 30 °C.

### *β*-Glucuronidase (Gus) assays

Cultures of *S. meliloti* 1021/pBB53Fsr::gusA were grown in MMSN minimal medium without or with three different concentrations of selected antibiotics, detergents or hydrogen peroxide (H_2_O_2_) for 16 h at 30 °C with shaking at 200 r.p.m. Gus activity was determined by measuring the hydrolysis of *p*-nitrophenyl *β*-d-glucuronide at 405 nm with quantification based on total protein [12] in two independent experiments with three technical replicates each. One unit (U) of activity is defined as the production of 1 nmol of product min^−1^ mg protein^−1^. Strain 1021 containing pBBR1MCS-53 without an insert lacked Gus activity.

### Swimming motility and biofilm formation assays

Swimming assays were done in Bromfield medium containing 0.3% Difco Noble Agar (Beckman, Dickinson and Co., Sparks, MD, USA) as described previously [11]. The diameters of the growth displacement zones were measured in triplicate assay, and their areas were calculated. Biofilm formation was determined by crystal violet (CV) staining as described in [11]. Biofilm formation was calculated as the A_595_ of the CV solutions divided by the OD_595_ of the cultures.

### Ethidium bromide accumulation and efflux assay

Ethidium bromide (EtBr) fluorescence assays were performed as described by Sharma *et al*. [18]. Cells from exponential phase cultures of *S. meliloti* 1021 and the *fsr* mutant 1021 Fsr were centrifuged at 13,000 ***g***, washed and resuspended in PBS to an OD₅₉₅ of 0.3. The EtBr accumulation assay was initiated by adding both EtBr and glucose to the cell suspension to final concentrations of 10 µg ml^−1^ and 0.4%, respectively. Because EtBr is passively taken up by Gram-negative bacteria, fluorescence measurements reflect the net balance between passive diffusion into the cells and active efflux. Note that since EtBr fluoresces more intensely when bound to nucleic acids inside the cell than when free in solution, the fluorescence signal predominantly reflects intracellular EtBr. Glucose provides an energy source for transporter-mediated efflux, which limits intracellular accumulation in strains with functional transporters. Six minutes after the start of the assay, the efflux inhibitor carbonyl cyanide m-chlorophenyl hydrazone (CCCP) was added to one of the reactions for each strain to a final concentration of 25 µg ml^−1^.

For the EtBr efflux assay, EtBr was first added to the cell suspension to a final concentration of 10 µg ml^−1^, and the sample was incubated at 30 °C for 15 min to allow the dye to accumulate inside the cells. During this incubation, EtBr binds intracellular nucleic acids, leading to increased fluorescence. Efflux was then initiated by the addition of glucose (0.4% final concentration), which supplies the energy required for active transporters to expel EtBr from the cells. Fluorescence was measured over time using a Qubit fluorometer with excitation and emission wavelengths of 470 and 610 nm, respectively. A decrease in fluorescence following glucose addition indicates active EtBr efflux, as the dye is transported out of the cells and diluted in the extracellular medium. Six minutes after the start of the assay, the efflux inhibitor CCCP was added to one of the reactions for each strain to a final concentration of 25 µg ml^−1^.

### Polyamine analysis

Endogenous polyamines in the *S. meliloti* 1021 wild type and *fsr* mutant were determined as dansyl chloride derivatives by high-performance TLC as described in [12].

### Preparation of alfalfa root exudates

To obtain alfalfa root exudates for *S. meliloti* growth inhibition assays, alfalfa seeds (var. Cuf101) were disinfected as described previously [19]. Six seeds were sown per Magenta jar (Fisher Scientific) containing vermiculite and configured as described in [20], with the reservoirs containing one-half-strength Fåhraeus nutrient solution [21] with or without nitrogen supplement. The units were incubated in a growth chamber maintained at 25 °C with 8/16 h dark/light periods. After 3 days, plantlets maintained with nitrogen-free or nitrogen-containing nutrient were mock uninoculated with 0.85% NaCl or were inoculated with 250 µl of *S. meliloti* Rm8530 cell suspension (~2.5×10^7^ c.f.u.) in 0.85% NaCl that was prepared with cells from an overnight PY culture. To obtain root exudates, six plants of each treatment were disinterred at 6, 17 and 38 dpi post-inoculation. The root portion of the plants was briefly rinsed in sterile distilled water to remove vermiculite and then was submerged in 5, 7 or 20 ml of sterile water contained in 6, 13 or 50 ml sterile plastic tubes, respectively, as appropriate for the volume of root tissue. Tubes were wrapped with aluminium foil, which was crimped around the plant stems protruding from the top of the tubes. Tubes were incubated in a growth chamber under the conditions stated above. After 24 h, fluid (root exudate) from the tubes was centrifuged at 13,000 ***g***, and the supernatants were stored at −70 °C. For assays, the samples were lyophilized to dryness, resuspended to 1 : 50 of their original volume in sterile water and centrifuged as above. Ten microlitres of aliquots of the supernatants were used in disc inhibition assays, in triplicate. Discs treated with sterile distilled water or fosfomycin (25 mg ml^−1^) served as negative and positive controls.

### Preparation of alfalfa seed exudate

Seed exudates for use in growth inhibition tests were prepared by a modification of a published protocol [22]. Briefly, 5 g of *Medicago sativa* cuf101 seeds was placed in a sterile 100 ml beaker and washed four times with 40 ml portions of sterile distilled water. Seeds were disinfected for 12 min with 40 ml H_2_O_2_ (3% v/v) and washed four times with sterile water. After draining off the liquid, 15 ml of sterile water was added, and the beaker was covered and left at 30° for 48 h. The fluid (seed exudate) obtained from the beaker was filter-sterilized and combined with an appropriately concentrated stock solution of MMSN minimal medium to grow cultures for *fsr* transcriptional fusion assays. For inhibition disc assays, the seed exudate was lyophilized to dryness and resuspended to 1 : 10 vol of sterile water and centrifuged as above. Ten microlitres of aliquots of the supernatants were applied to discs, in triplicate.

#### *M. sativa*–*S. meliloti* symbiosis assays

Two-day-old *M. sativa* (var. Cuf101) seedlings prepared as described previously [19] were transferred to pots with sterile vermiculite and inoculated with 200 µl of an *S. meliloti* overnight culture grown in PY. Plants were watered every third day with a nitrogen-free nutrient solution, alternating with water. Plants were grown in a greenhouse at 25–28 °C and harvested 45 days after inoculation. Nitrogenase activity and nodule and plant dry weights were determined as described in [19] from three independent experiments.

## Results

### Sequence analysis shows that Fsr is a single-component MFS transporter

The structures of most MFS members have 12 transmembrane sections surrounding a central cavity, but some have up to 16 transmembrane sections [23]. The 398 amino acid protein encoded by the *S. meliloti* 1021 *smc00990* gene is annotated as an MFS family (Prosite PS50850) putative fosmidomycin resistance transmembrane protein, or Fsr. The secondary structure of Fsr proteins in the AlphaFold and Uniprot database (accession Q92RK8) has 11 helical transmembrane segments (Fig. S1A, available in the online Supplementary Material), whilst the Deep TMHMM [24] prediction shows 12 transmembrane segments (Fig. S1B).

The NCBI Conserved Protein Domain Family database (accession cd17478) provides a consensus sequence that includes 35 residues predicted to be involved in substrate binding in the MFS fosmidomycin resistance proteins (https://www.ncbi.nlm.nih.gov/Structure/cdd/cddsrv.cgi?uid=cd17478). Alignment of the *S. meliloti* 1021 Fsr with the consensus shows that 18 of 35 (51%) of the predicted substrate-binding residues are identical in the *S. meliloti* Fsr, and 28 residues (80%) are identical or substituted with a similar amino acid (Fig. S2). The *S. meliloti* Fsr is 54.8% identical to the Fsr fosmidomycin efflux protein of *E. coli* K-12 [25] but only 22.8% identical to *Acinetobacter baumannii* ATCC 17978 AbaF protein, which encodes an efflux protein for the fosmidomycin analogue fosfomycin [18].

Fsr from strain 1021 is over 90% identical to the majority of the corresponding proteins in other *Sinorhizobium* and *Ensifer* strains, which share the same *fsr-nspS-mbaA* gene context. Fsr proteins in some other rhizobia are plasmid-encoded, for example, in *Rhizobium leguminosarum* bv. *viciae* 3841 and *R. etli* CE3. These latter Fsr proteins share just over 75% sequence identity with the *S. meliloti* Fsr, and over 95% identity with one another.

### Fsr increases resistance to some antimicrobial compounds

We used disc inhibition assays to determine how the antimicrobial sensitivity of the 1021 *fsr* null mutant (strain 1021 Fsr) differed from that of the wild type. To confirm the requirement for the *fsr* gene in conditioning the mutant’s phenotypes, we also assayed the *fsr* mutant containing the *fsr* gene borne on plasmid pFAJ1700 [strain 1021 Fsr (pFAJ_Fsr)] as well as the mutant containing the pFAJ1700 vector without an insert [1021 Fsr (pFAJ1700)].

Contrary to our expectations, given the annotation of *fsr* as encoding a fosmidomycin efflux pump, both the 1021 wild type and *fsr* mutant exhibited equally high resistance to fosmidomycin. They formed only small zones of inhibition in the disc assay (Table 2) and had fosmidomycin MICs exceeding 256 mg ml⁻¹ in the broth microdilution assay (Table 3).

**Table 2.** Sensitivity of the *S. meliloti* 1021 WT and *fsr* mutant (1021 Fsr) strains* to antimicrobial agents. Values represent the diameter (mm) of the zone of inhibition^†^ for each antimicrobial agent tested

	Strain			
	1021	1021 Fsr	1021 Fsr (pFAJ1700)*	1021 Fsr (pFAJ_Fsr)*
**Antimicrobial agent**	**Diameter (mm) of the zone of inhibition in the presence of†**			
Fosfomycin	13.6±3.1	68.6±4.5‡	57.7±1.5‡	21.7±2.1‡
Fosmidomycin	2.5±0.2	2.5±0.2	2.5±0.1	2.4±0.2
Chloramphenicol	52.0±2.0	54.7±1.5	53.7±1.5	55.0±1.0
Neomycin	32.8±3.6	26.3±3.2	26.7±2.3	35.3±3.5
Kanamycin	33.0±3.0	35.2±2.3	37.7±3.4	37.0±6.2
Deoxycholic acid	9.8±2.5	16.2±2.4‡	15.3±1.5‡	11.3±1.2
H_2_O_2_	20.5±2.5	31.4±4.4‡	35.4±3.7‡	25.7±4.2
Salicylic acid	13.3±2.3	13.7±0.6	14.0±1.0	15.3±1.5
Citric acid	19.0±1.7	18.7±0.6	18.7±0.6	20.0±1.7
SDS	17.2±2.6	8.8±1.0‡	9.7±2.5‡	21.0±1.0

*The pFAJ1700 lacks an insert, and pFAJ_Fsr contains the *fsr* gene as an insert.

†Mean±sd of inhibition zones obtained from four independent experiments (*n*=4).

‡Values that are significantly different (*P*<0.05) from the corresponding value for WT strain 1021.

**Table 3.** MIC determination assays. Values are the MIC of antimicrobial agent obtained in three independent experiments, expressed in µg ml^−1^

Antimicrobial agent	**1021**	1021 Fsr	Fsr (pFAJ1700)	Fsr (pFAJ_Fsr)
Fosfomycin	>256	0.125	0.125	>256
Fosmidomycin	>256	>256	>256	>256
Kanamycin	8	8	8	8
Gentamicin	4	4	4	4
Ciprofloxacin	<0.012	<0.012	<0.012	<0.012
Neomycin	0.5	0.5	0.5	0.5
Chloramphenicol	0.06	0.03	0.03	0.06
Nalidixic acid	>256	>256	>256	>256
Rifampin	0.5	0.5	0.5	0.5

Fosmidomycin targets the enzyme Dxr of the non-mevalonate isoprenoid synthesis pathway [26]. Fosmidomycin resistance in *S. meliloti* does not appear to be due to its lacking the antibiotic’s target enzyme: gene *smc03105* is annotated as encoding Dxr, which catalyses the second step of the non-mevalonate isoprenoid pathway (https://www.genome.jp/module/sme_M00096). Tn-seq analysis showed that smc03105 was an essential gene in *S. meliloti*, consistent with its participationg in the sole pathway for isoprenoid synthesis present in this species [27].

The ability of *S. meliloti* 1021 to take up fosmidomycin was determined using zone inhibition assays with *E. coli* DH5α as the indicator strain. In these assays, supernatants obtained from cultures of *S. meliloti* 1021 and *E. coli* DH5α that had been incubated with fosmidomycin (1 mg ml^−1^) formed, respectively, 4.0±0.8 mm and 5.7±1.2 mm growth inhibition zones in the *E. coli* DH5α indicator strain. The lysate prepared from cells from the fosmidomycin-treated culture of strain DH5α formed a 5.0±0.8 mm inhibition zone, whilst the lysate from strain 1021 caused no inhibition of the indicator strain. This indicates that the high resistance of strain 1021 to fosmidomycin may be due to a lack of antibiotic uptake.

For the antimicrobial compounds included in our screening, inactivation of *fsr* caused the greatest increase in sensitivity to fosfomycin. The fosfomycin inhibition zone for the *fsr* mutant was five times larger than that of the wild type (Table 2). The fosfomycin inhibition zone of the 1021 Fsr (pFAJ1700) empty vector strain was 2.7-fold larger than that of the *fsr*-complemented mutant 1021 Fsr (pFAJ_Fsr), indicating partial complementation of the mutant when *fsr* was expressed *in trans*. Consistent with the sensitivity of *S. meliloti* to fosfomycin, the strain 1021 MurA protein (SMc02305; UDP-*N*-acetylglucosamine 1-carboxyvinyltransferase) conserves a Cys residue at position 126 that corresponds to the Cys115 residue that is targeted by the antibiotic in the *E. coli* MurA, an enzyme essential for peptidoglycan synthesis [28].

Based on the inhibition zone diameters, the *fsr* mutant was 1.5- and 1.7-fold more sensitive to inhibition by H_2_O_2_ and deoxycholate (DOC), respectively, in comparison to the wild type (Table 2). Resistance to these compounds was largely restored to WT levels in the *fsr*-complemented mutant. The *fsr* mutant was 50% less sensitive to inhibition by SDS versus the wild type. The introduction of the plasmid with *fsr* into the mutant increased its sensitivity to SDS to a level similar to that of the wild type (Table 2).

The growth of the *fsr* mutant in the presence of neomycin, kanamycin, chloramphenicol, salicylic or citric acid was not statistically different from that of the wild type. We anticipated that in comparison to the wild type, alfalfa seed or root exudates might be more inhibitory to the *fsr* mutant’s growth due to its reduced ability to expel the compounds. However, neither seed nor root exudates inhibited the growth of either strain in the disc assays using concentrated exudate preparations obtained as described in the Methods section. Discs to which sterile water was applied likewise caused no inhibition, whilst discs containing fosfomycin (10 µl of a 1 mg ml^−1^ solution) formed inhibition zones with areas similar to those shown in Table 2 for strain 1021.

The MIC of several antibiotics for strain 1021, the *fsr* mutant and its plasmid-containing derivatives were determined by broth dilution assay (Table 3). Both the 1021 wild type and *fsr* mutant were equally and highly resistant to fosmidomycin (MIC>256 µg ml^−1^; Table 3). The *fsr* mutant with or without empty vector pFAJ1700 was 2,000 times more sensitive to fosfomycin than the wild type or the mutant complemented with the *fsr* gene (Table 3). For chloramphenicol, the *fsr* mutant without or with the empty cloning vector had an MIC half that of the wild type or the mutant containing the cloned *fsr* gene. The MICs for kanamycin, gentamicin, ciprofloxacin, neomycin, nalidixic acid and rifampin were identical for the wild type, and for the *fsr* mutant without plasmid, with the empty vector or with the plasmid-borne *fsr* gene.

**Fig. 1.**
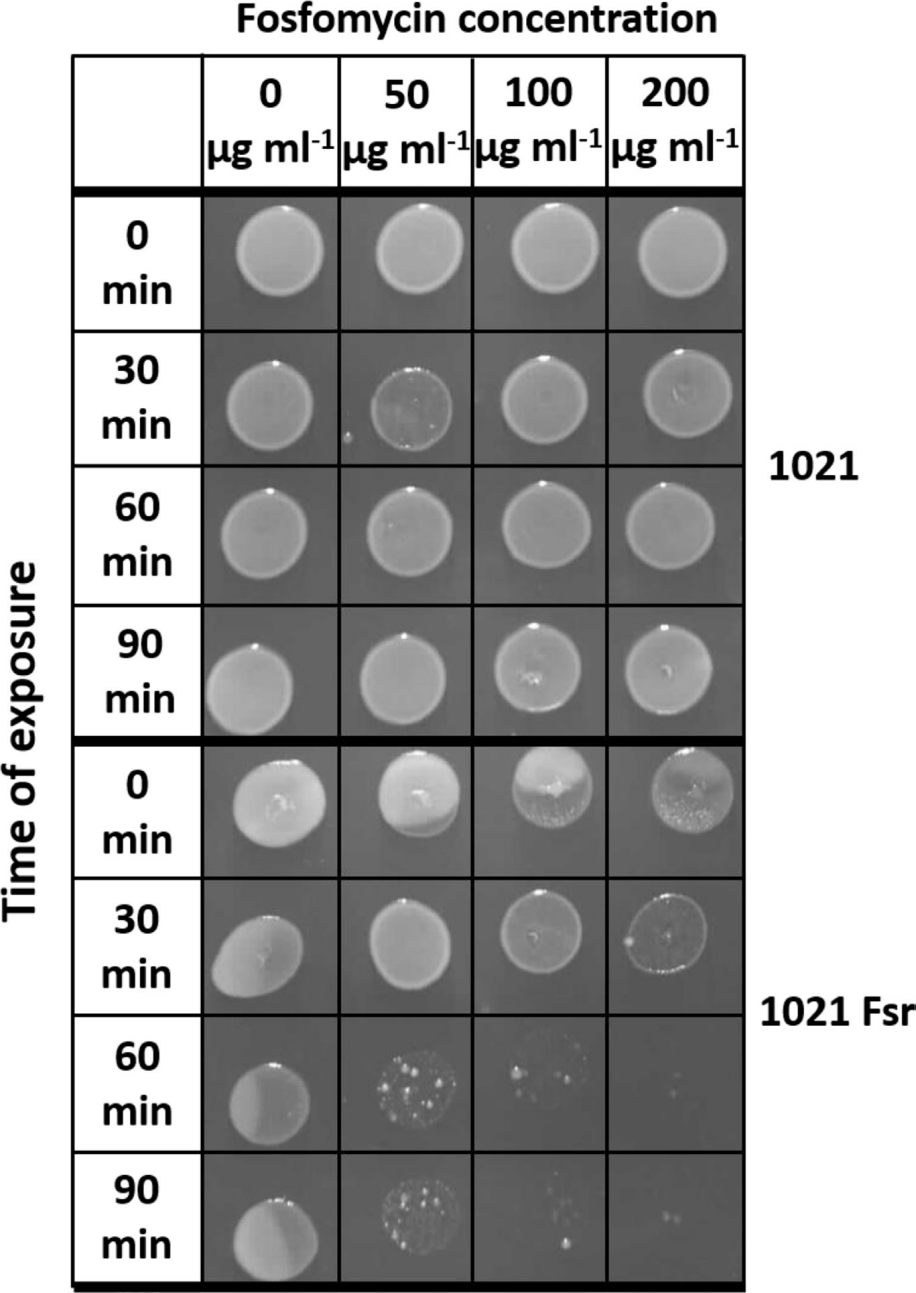
Effect of fosfomycin on the growth of *S. meliloti* 1021 wild type and *fsr* mutant 1021 Fsr. Cells in the exponential phase were transferred to PY medium containing 0, 50, 100 or 200 µg ml^−1^ fosfomycin. Ten-microlitre aliquots were removed at 0, 30, 60 and 90 min, spotted onto PY medium and incubated at 30 °C as described in the Methods section.

The great difference in fosfomycin sensitivity between the *fsr* mutant and the wild type was confirmed with a drop-plate assay in which cultures were exposed to different concentrations of fosfomycin for different time periods and spotted onto rich medium (Fig. 1). Forty-eight hours after spotting, the growth of the wild type exposed to even the highest fosfomycin concentration (200 µg ml^−1^) for 90 min was not inhibited. In contrast, the growth of the *fsr* mutant was drastically reduced by exposure to the lowest fosfomycin concentration (50 µg ml^−1^) for 60 or 90 min.

### Fluorimetric assays with EtBr show that Fsr is an efflux pump

**Fig. 2.**
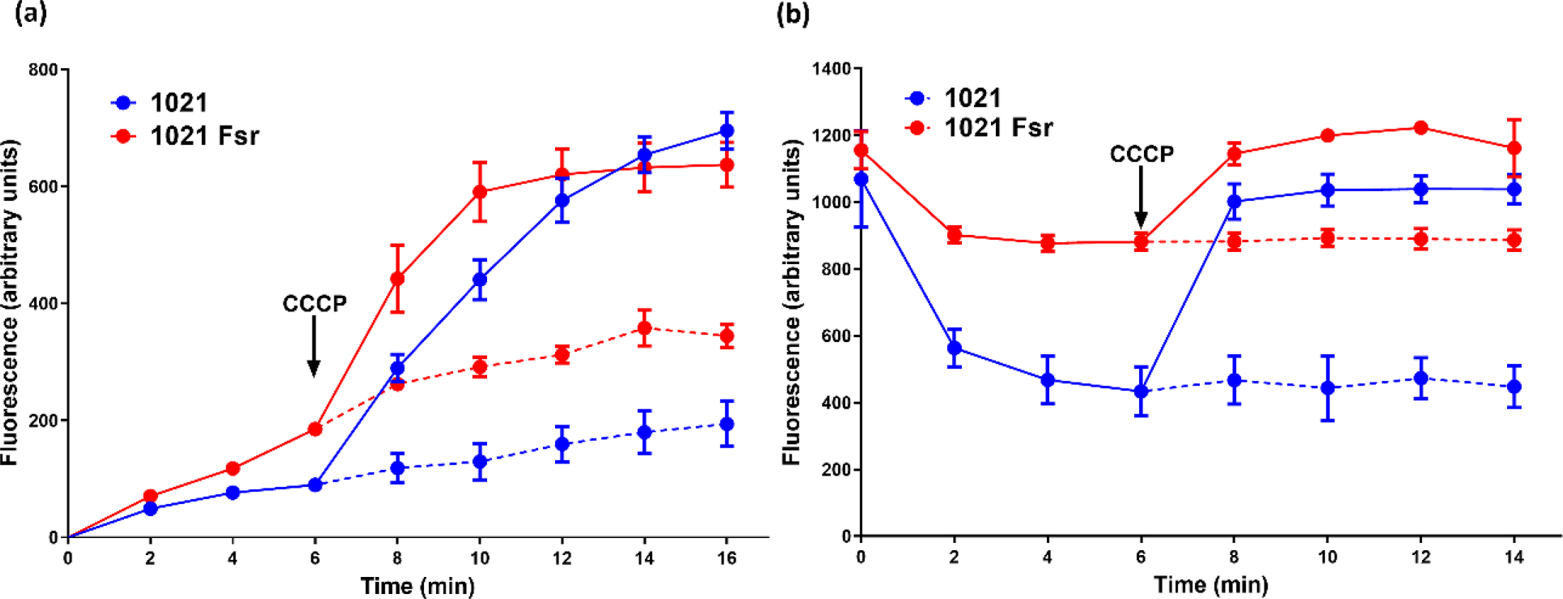
(**a**) EtBr accumulation in *S. meliloti* 1021 (blue lines) and *fsr* mutant 1021 Fsr (red lines) in the presence of glucose was determined using a fluorometer. (**b**) To measure EtBr efflux, *S. meliloti* 1021 (blue lines) and 1021 Fsr (red lines) cells were incubated in a medium with EtBr to allow loading of the cells with this compound. Glucose was added to the medium to initiate efflux of EtBr. The efflux inhibitor CCCP was added to the mixture at the timepoint indicated. The dotted lines represent the relative fluorescence in assays without CCCP. The experiment was repeated four times using two replicates (*n*=8). Error bars indicate sd.

EtBr is passively taken up by Gram-negative bacteria [29] and is used as a substrate in efflux assays of efflux pumps. We used EtBr in fluorometric efflux and uptake assays to determine if Fsr activity was dependent on an electrochemical gradient. The uptake assays showed that EtBr accumulation was clearly lower in WT 1021 than in the *fsr* mutant (Fig. 2a), consistent with Fsr allowing a more efficient expulsion of the compound from the WT cells. The addition of the energy uncoupler and efflux inhibitor CCCP caused similar rapid increases in the rate of EtBr accumulation in both WT and mutant strains, consistent with all proton-dependent efflux proteins capable of EtBr expulsion being inhibited.

In the EtBr efflux assays (Fig. 2b), cells of the wild type and *fsr* mutant were loaded with EtBr, and efflux was initiated by adding glucose. The rate and extent of EtBr efflux were higher in the wild type as compared to the mutant. The addition of CCCP caused an abrupt increase in fluorescence, the magnitude of which was greater in the wild type (containing an active Fsr prone to CCCP inactivation) as compared to the mutant, in which Fsr was inactivated (Fig. 2b).

### Fosfomycin induces *fsr* expression

**Fig. 3.**
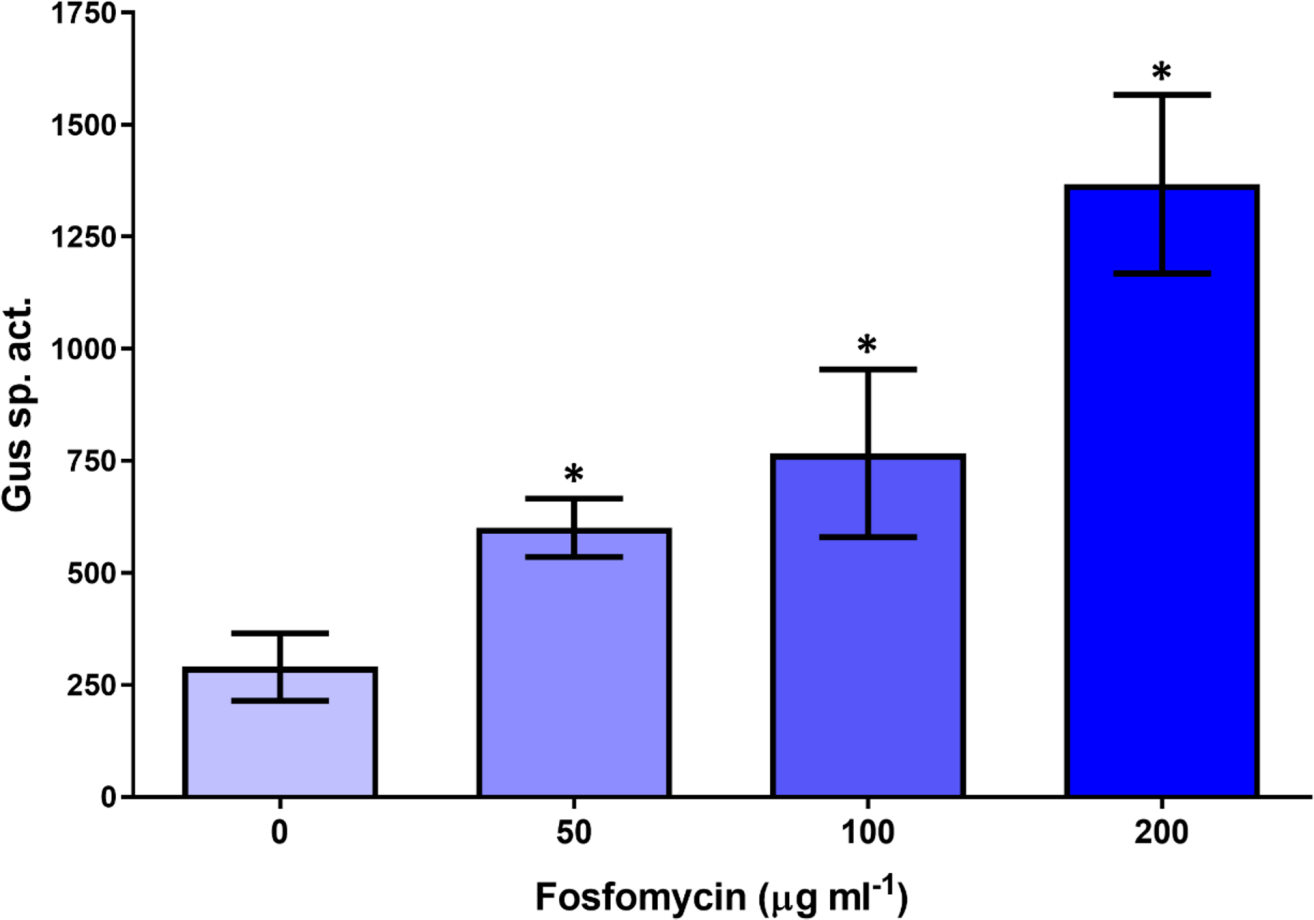
*β*-Glucuronidase (Gus) activity produced by *S. meliloti* 1021 containing the transcriptional fusion plasmid pBB53Fsr::gusA grown for 18 h in MMSN with the indicated concentration of fosfomycin. Gus specific activity is in nmol min^−1^ mg protein^−1^. Values are the mean±sd for two independent experiments with three technical replicates. One-way ANOVA with a Tukey post-test was performed, and asterisks indicate treatments differing from the control with *P*<0.05.

We monitored *fsr* expression in strain 1021 containing a plasmid with a *fsr-gusA* (*uidA*) transcriptional fusion. Using this construct, we found that the transcriptional expression of Fsr increased 2.0-, 2.6- and 4.7-fold in *S. meliloti* cells treated with 50, 100 and 200 µg ml^−1^ fosfomycin, respectively, as compared with its expression in cells grown without antibiotic (Fig. 3). In contrast, the expression of *fsr* in cultures treated with different concentrations of chloramphenicol, kanamycin, H_2_O_2_, SDS and DOC did not change with respect to control cultures lacking these compounds (Fig. S3). *fsr* transcription increased nearly 1.6-fold when the fusion strain was grown in the presence of 50% (v/v) alfalfa seed exudate in medium MMSN (Fig. S3).

### Biofilm formation and motility are affected in the *fsr* mutant

Because efflux pump activity affects biofilm formation and motility in some bacteria, and because *fsr* lies next to *nspS* and *mbaA* encoding a polyamine sensing system that modulates these phenotypes, we assayed biofilm formation and swimming motility in the 1021 *fsr* mutant.

**Fig. 4.**
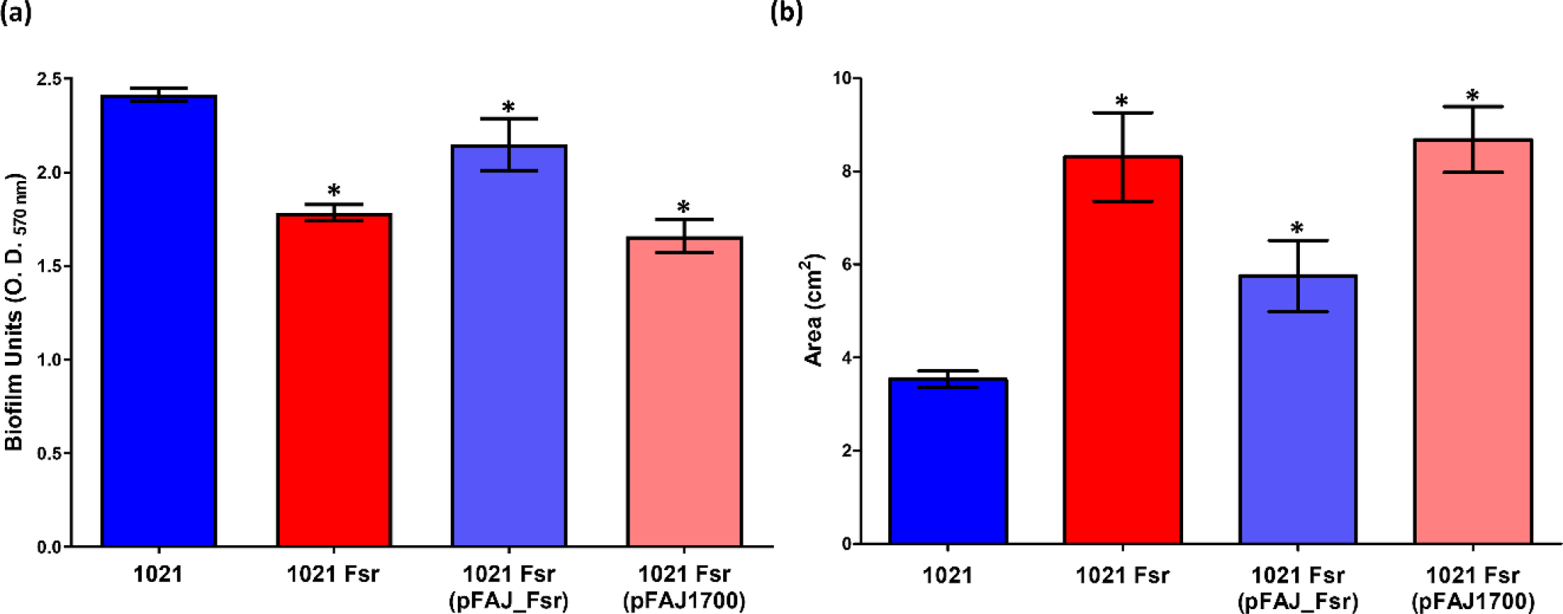
Biofilm formation (**a**) and swimming motility (**b**) of *S. meliloti* 1021, 1021 Fsr, 1021 Fsr (pFAJ_Fsr) and 1021 Fsr (pFAJ1700). Biofilm was determined by CV staining of cultures grown in borosilicate glass tubes. The experiments were performed in quadruplicate and repeated three times. Error bars represent sd. One-way ANOVA with a Tukey post-test was performed, and asterisks indicate treatments differing from the control with *P*<0.05.

To determine the effect of *fsr* inactivation on biofilm formation, we grew the *S. meliloti* strains in minimal medium and determined biofilm formation after 3 days (Fig. 4a). The *fsr* mutant without or with vector pFAJ1700 produced 25% less biofilm than the wild type. Biofilm formation by the *fsr* mutant genetically complemented with plasmid pFAJ_Fsr was only 11% lower in comparison to strain 1021, indicating partial complementation by the *fsr* expressed *in trans*.

We evaluated swimming motility by calculating the areas of the growth displacement zones of strains that had been stab-inoculated in plates of a semisolid medium and incubated for 3 days. Compared to the wild type, swimming motility was 2.3- and 2.5-fold higher in the *fsr* mutant without or with an empty vector, respectively (Fig. 4b). Strain 1021 Fsr (pFAJ_Fsr) had swimming motility intermediate between that of the wild type and the *fsr* mutant, consistent with partial complementation by the plasmid-borne *fsr*.

We showed previously that a strain 1021 *nspS* null mutant formed more biofilm and had reduced swimming ability in comparison to the wild type [11]. These phenotypes are the opposite of those of the *fsr* mutant, described above, but do not result from differences in endogenous polyamine levels between the mutant and wild type (Fig. S4).

### Fsr mutation reduces the efficiency of symbiosis with alfalfa

**Fig. 5.**
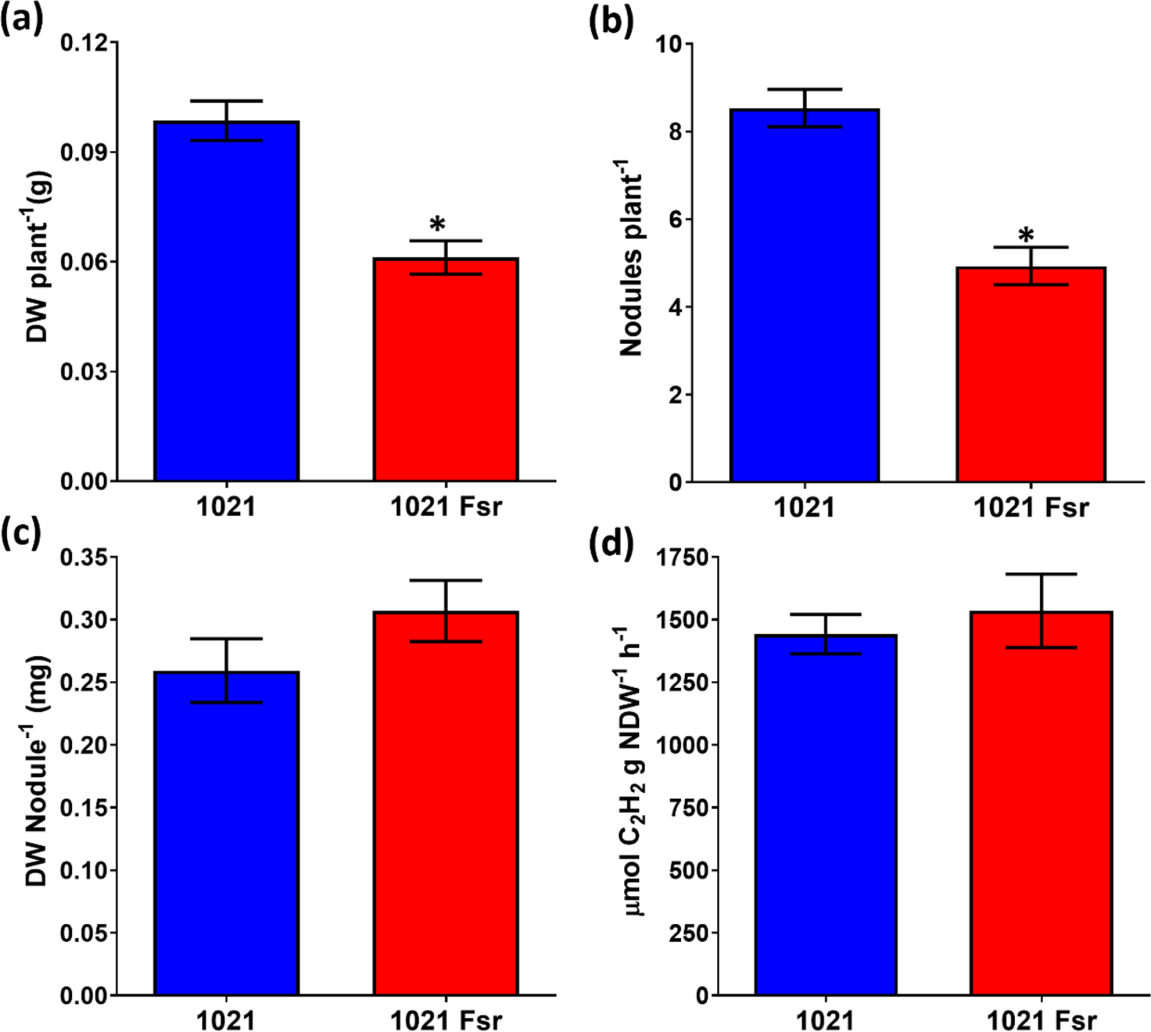
Symbiotic parameters of *S. meliloti* 1021 and 1021 Fsr with alfalfa. (**a**) Plant dry weight. (**b**) Number of nodules per plant. (**c**) Nodule biomass. (**d**) Acetylene reduction activity per gram dry weight of nodules. Values are the mean±sd for three independent experiments. Asterisks indicate treatments differing from the control with *P*<0.05 according to a Tukey post-test.

To examine the role of Fsr in symbiosis, WT 1021 and the *fsr* mutant were individually inoculated onto alfalfa. Forty-five days after inoculation, the dry weight of plants and the number of nodules per plant were 38 and 42% less for the plants inoculated with the *fsr* mutant in comparison to the wild type, respectively (Fig. 5a, b). The acetylene reduction specific activity and nodule dry weights of the plants inoculated with the mutant were not statistically significantly different than those of the wild type (Fig. 5c, d).

### The *fsr* mutant produces WT levels of endogenous polyamines

We noted above the opposing effects on biofilm formation and swimming motility in the *fsr* and *nspS* mutants compared to the WT strain: the *fsr* mutant formed less biofilm and exhibited increased swimming motility, whereas the *nspS* mutant formed more biofilm and showed reduced motility. Because changes in endogenous polyamine levels affect biofilm formation, motility and symbiotic efficiency in *S. meliloti* [30], we analysed the free polyamine content of strains 1021 and the *fsr* mutant grown in culture. The polyamine profiles of both strains were identical and consisted of putrescine, spermidine and homospermidine (Fig. S4), as expected from previous results [12].

## Discussion

We previously described the role of the NspS-MbaA sensor/transducer signalling system that affects biofilm formation, motility and symbiotic efficiency in *S. meliloti* in response to exogenous polyamines ([11] and results not shown). The *fsr* gene immediately precedes *nspS* in virtually all sequenced *Sinorhizobium* species [11]. Because Fsr, like some other antibiotic exporters, might function not only in cellular detoxification but also in conditioning other phenotypes [31], we undertook the present study to determine some of its characteristics.

The *S. meliloti* 1021 Fsr protein is annotated as a putative fosmidomycin resistance transmembrane protein. Fsr is predicted to contain 11 or 12 transmembrane alpha-helical domains, depending on the computational programme used for their detection (Figs S1A and S1B). Both of these transmembrane arrangements occur amongst MFS family members, although the functional significance of 11 versus 12 transmembrane segments is unknown [5].

Contrary to our expectations, both the *S. meliloti* wild type and *fsr* mutant showed a high level of resistance to fosmidomycin, despite containing the *dxr* gene that encodes the antibiotic’s target. Bacteria including *E. coli*, *Francisella novicida*, *Mycobacterium tuberculosis* and *Brucella abortus* transport fosmidomycin using the glycerol-3-phosphate transporter [17,32, 33]. Our transport assays with fosmidomycin indicated that *S. meliloti* 1021 does not transport a detectable level of the antibiotic despite having a constitutively expressed glycerol 3-phosphate uptake system (UgpBAEC) encoded in its genome [34]. It is possible that *S. meliloti* can take up fosmidomycin but either inactivates it or expels it using another efflux system.

The greatly increased sensitivity of the *S. meliloti fsr* mutant to fosfomycin shows that Fsr is essential for resistance to this antibiotic. To a lesser extent, Fsr increases the resistance of *S. meliloti* to the detergent DOC and the oxidizing agent H_2_O_2_ (Tables2  3). However, the fact that *fsr* expression increased only in the presence of fosfomycin, but not DOC or H_2_O_2_, indicates a specific induction of the gene in response to the antibiotic (Figs 3 and S3).

Growth inhibition assays with alfalfa seed and root exudates showed that neither exudate inhibited the wild type or the *fsr* mutant, indicating that Fsr is not involved in avoiding toxic compounds from alfalfa. However, exposure of *S. meliloti* to fosfomycin could take place in the soil, where *Streptomyces* and other producers of the antibiotic also reside [35]. A role for Fsr in the soil or in early stages of the symbiosis is consistent with the finding that *fsr* expression was reduced 4- to 5-fold in bacteroids of both WT and *fixJ* mutant strains of *S. meliloti* 1021 isolated from *Medicago truncatula* nodules, compared to cells grown in rich medium [36]. This down-regulation of fsr expression in bacteroids, independent of FixJ – a key regulator active in the later stages of symbiosis – suggests that Fsr functions primarily outside the nodule or before bacteroid differentiation.

The functionality of Fsr as an efflux pump was shown by the reduced ability of the *fsr* mutant to take up and expel EtBr relative to the WT strain (Fig. 2). The effects of the electrochemical gradient uncoupler CCCP on efflux and uptake indicate that the Fsr is an electrochemical gradient-dependent efflux transporter.

Our findings support a role for Fsr in EtBr efflux, as shown by the reduced accumulation in the wild type compared to the *fsr* mutant (Fig. 2a). However, other factors in addition to active efflux could contribute to the observed differences. Notably, EtBr loading appears similar between strains (Fig. 2b), suggesting comparable passive uptake. However, the increased resistance of the *fsr* mutant to DOC and H_₂_O_₂_, together with the lack of *fsr* induction by these agents, raises the possibility that differences in membrane permeability or envelope structure could affect EtBr retention or overall cell physiology. These broader changes could also contribute to altered motility and biofilm formation.

An intact Fsr system positively affects biofilm formation, since disrupting *fsr* decreased biofilm formation in strain 1021 (Fig. 4a). Specific efflux pumps have been reported to increase biofilm formation in ESKAPE pathogens, where they function to extrude biofilm matrix components or quorum sensing molecules [18,37,40].

Swimming motility was significantly increased in the *fsr* mutant in comparison to the WT strain (Fig. 4b). The reciprocal changes in motility and biofilm formation in the *fsr* mutant could ultimately be caused by an effect of the Fsr efflux system on quorum-sensing and/or intracellular cyclic di-GMP levels [22,41]. This hypothesis requires experimental confirmation.

Biofilm formation, motility and stress resistance in the microsymbiont are important factors for the establishment of the * S. meliloti*–alfalfa symbiosis [30]. Symbiosis assays show that the *fsr* mutant formed nodules with WT nitrogenase activity, but the nodule number and the dry weight of the plants were significantly lower in the mutant. This indicates that colonization may be compromised in the mutant, possibly because of its lower biofilm production and greater susceptibility to oxidative stress.

In conclusion, the *S. meliloti* 1021 Fsr system is an electrochemical gradient-dependent efflux pump important for resistance to a number of antimicrobial compounds, including fosfomycin. Inactivation of *fsr* in strain 1021 increased swimming motility and decreased biofilm formation and symbiotic efficiency with alfalfa.

## Supplementary material

10.1099/mic.0.001566 Uncited Supplementary Material 1.

## Abbreviations

CCCP: carbonyl cyanide m-chlorophenyl hydrazone
CV: crystal violet
DOC: deoxycholate
Dxr: 1-deoxy-d-xylulose-5-phosphate reductoisomerase
EtBr: ethidium bromide
H_2_O_2_: hydrogen peroxide
LB: Luria broth
MDR: multidrug resistance
MFS: major facilitator superfamily
MMSN: minimal medium succinate-ammonium
PY: peptone-yeast extract
RND: resistance-nodulation-cell division
