# Supplementary material for: The Fsr transporter of Sinorhizobium meliloti contributes to antimicrobial resistance and symbiosis with alfalfa
Source: Microbiology (Reading). 2025 May 21;171(5):001566. doi: 10.1099/mic.0.001566 (PMC12095868; doi:10.1099/mic.0.001566)
Supplement: Uncited Supplementary Material 1. [file mic-171-01566-s001.pdf]

Supplementary material

The major facilitator superfamily (MFS) transporter Fsr is required for fosfomycin resistance and contributes to symbiotic efficiency in *Sinorhizobium meliloti* 1021

Víctor M. Chávez-Jacobo*[1], Alma R. Reyes-González[2], Lourdes Girard[3] and Michael F. Dunn*[2]

[1]Departamento de Microbiología Molecular, Instituto de Biotecnología, Universidad Nacional Autónoma de México, Avenida Universidad 2001, Cuernavaca, Morelos, México.

[2]Programa de Genómica Funcional de Procariotes and [3]Programa de Microbiología Genómica, Centro de Ciencias Genómicas, Universidad Nacional Autónoma de México, Avenida Universidad s/n, Cuernavaca, Morelos, México.

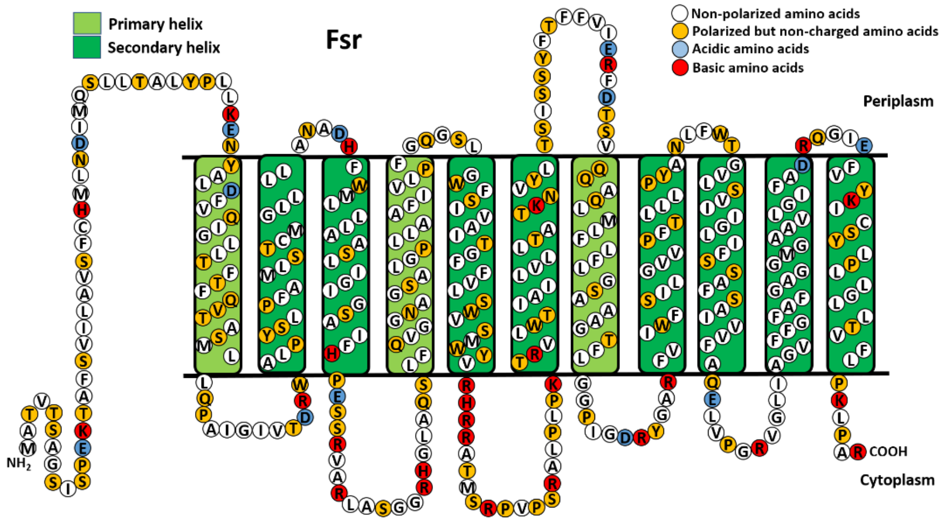

Figure S1A. Predicted secondary structure of Fsr. The secondary structure of Fsr was predicted from the sequence information using AlphaFold protein structure database (https://alphafold.ebi.ac.uk/search/text/Q92RK8). The protein contains 11 transmembrane helices.

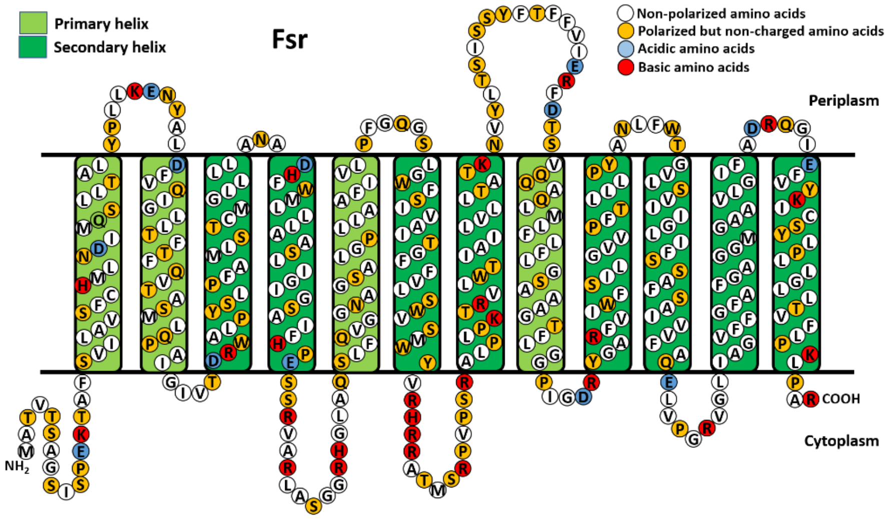

Figure S1B. Predicted secondary structure of Fsr. The secondary structure of Fsr was predicted from the sequence information using HMMTOP (http://www.enzim.hu/hmmtop/html/submit.php). The protein contains 12 transmembrane helices that are typical of MFS transporters.

```
consensus ----------------------LSLGHFLNDFYQGFLPALLPLLIAKLGLSYAQAGLLVS  38
1021 Fsr  MATVTSAGSISPEKTAFSVILAVSFCHMLNDIMQSLLTALYPLLKENYALDFVQIGLLTF  60
                                :*: *:***: *.:* ** ***  : .*.:.* ***.

consensus VFSITSSLLQPLFGYLADRKPRRYLLPLGPLLTGLGLSLLGFAPNYWLLLLLVALAGIGS  98
1021 Fsr  TFQVTASMLQPAIGIVTDRWALPYSLPFAMLSTCMGLLLLANADHFWMLLLAASLIGIGS  120
          .*.:*:*:*** :* ::**    * **:. * * :** **. * ::*:*** .:* ****

consensus AAFHPEGARLASQAAGGRRGLAMSIFSVGGNLGFALGPLIAAALVALFGGL--GLLILLI  156
1021 Fsr  AIFHPESSRVARLASGGRHGLAQSLFQVGGNAGSALGPLLAAFIVLPFGQGSLGWFSIVA  180
          * ****.:*:*  *:***:*** *:*.**** * *****:** :*  **    * : ::

consensus IPGLAVALL----LLRLR--PLKKPEAKKAAAKSSARSTLKGVWRALLLLLIIVFLRSWV  210
1021 Fsr  ITGFFVLSWVSMWYVRHRRATMSRPVPS----RALPLPKTRVLWTIAILVLLT-ATKNVY  235
          * *: *        :* *   :.:*  .    ::    . : :*   :*:*:    :.

consensus LSSLTTFLPFYLVQRGY-SLVFAGlLLSLFLFAGAIGTLIGGPLSDRFGRRRVIIASLLL  269
1021 Fsr  LTSISSYFTFFVIERFDTSVQQAQLMLFLFLGSAAAGTFLGGPIGDRYGARFVIWFSILG  295
          *:*::::: *::::*   *:  * *:* *** :.* **::***:.**:* * **  *:*

consensus AAPFLLLFLLTPGIWSYILLVLAGFLLFASFPVTVVLAQELLPGNVGLASGLMMGLSFGL  329
1021 Fsr  VVPFTLLLPYANLFWTGVLSVIIGLIFSSAFSAIVVFAQELVPGRVGLIAGVFFGFAFGA  355
          ..** **:  :  :*: :* *: *::: ::* . **:****:**.*** :*:::*::**

consensus GGIGAPVLGALADAFGLESVLLILALLPLLGALLAF------- 365
1021 Fsr  GGMGAAVLGIFADRQGIEFVYKICSYLPLLGLLTVFLPKLPAR 398
          **:** *** :**  *:* *  * : ***** * .*
```

Fig. S2. Alignment of the *S. meliloti* 1021 Fsr amino acid sequence with the Major Facilitator Superfamily fosmidomycin resistance protein consensus sequence showing residues predicted to be involved in substrate binding. The consensus sequence was taken from the NCBI Conserved Protein Domain Family database (accession cd17478), generated by aligning the *E. coli* K-12 Fsr protein with several bacterial drug-export proteins whose sequences identify them as Fsr proteins. The alignment is available under (https://www.ncbi.nlm.nih.gov/Structure/cdd/cddsrv.cgi?uid=cd17478). The consensus sequence containing 35 residues predicted to be involved in substrate binding was aligned with the 1021 Fsr sequence using Clustal Omega. Predicted substrate binding residues are shown in bold green letters in the consensus sequence; exact matches in the 1021 Fsr sequence are highlighted likewise.

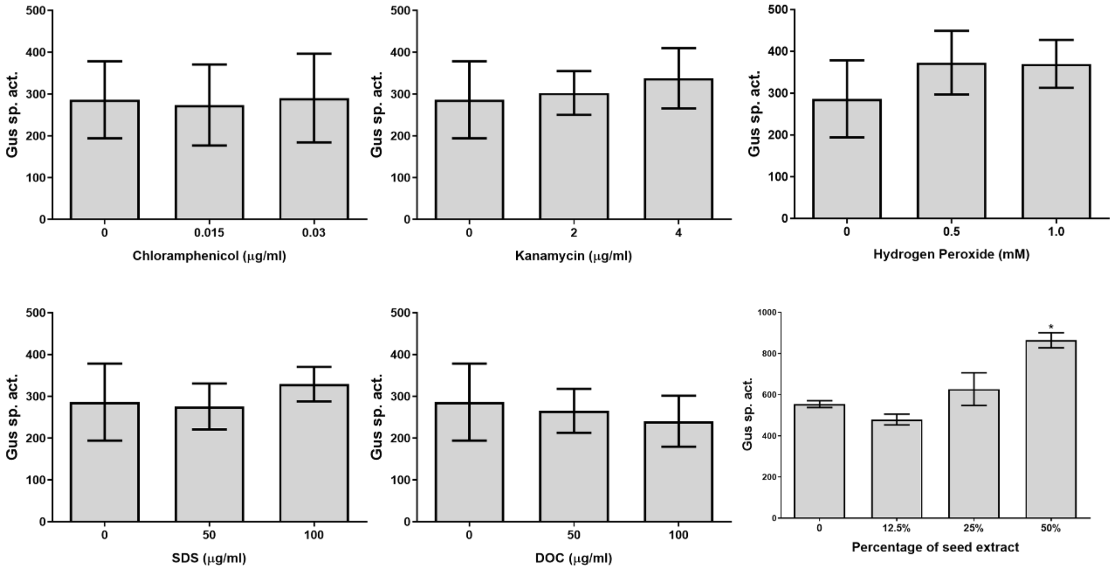

Figure S3. β-glucuronidase (Gus) activity produced by *S. meliloti* 1021/pBBR53Fsr::gusA grown for 18 h in MMSN minimal medium with the indicated concentration of indicated compound. Values are the mean+SD for two independent experiments with three technical replicates.

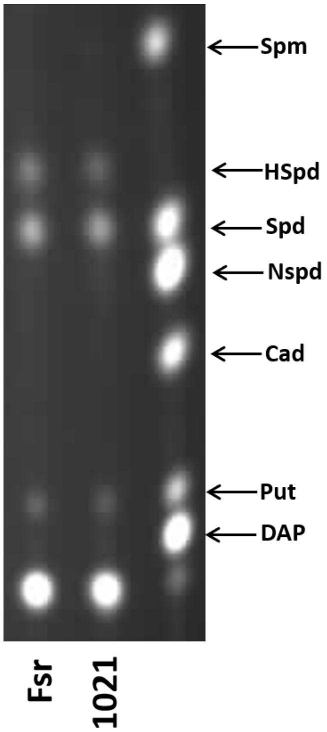

Figure S4. HPTLC detection of cytoplasmic dansyl-polyamines from 24 h cultures 0f the S. meliloti 1021 wild type and Fsr mutant. Dansyl-polyamine identities are shown to the right of the image (1,3-diaminopropane (DAP); putrescine (Put); cadaverine (Cad); norspermidine (Nspd); spermidine (Spd); homospermidine (HSpd); and spermine (Spm)). The polyamine profiles of both strains are identical, consisting of putrescine, spermidine, and homospermidine.
